# Comparison in Short-Term Safety and Efficacy between New-Generation WATCHMAN FLX and Conventional WATCHMAN 2.5 for Percutaneous Left Atrial Appendage Closure

**DOI:** 10.3390/jcm11061618

**Published:** 2022-03-15

**Authors:** Nobuyuki Fukuda, Teruhiko Imamura, Shuhei Tanaka, Naoya Kataoka, Ryuichi Ushijima, Hiroshi Ueno, Koichiro Kinugawa

**Affiliations:** The Second Department of Internal Medicine, University of Toyama, 2630 Sugitani, Toyama 930-0194, Japan; nfukuda@med.u-toyama.ac.jp (N.F.); stanaka@med.u-toyama.ac.jp (S.T.); nkataoka@icloud.com (N.K.); ryuryu0702@gmail.com (R.U.); hueno@med.u-toyama.ac.jp (H.U.); kinugawa-tky@umin.ac.jp (K.K.)

**Keywords:** atrial fibrillation, anti-coagulation, thrombosis

## Abstract

Background: Percutaneous left atrial appendage closure using the WATCHMAN system, to prevent thrombosis and minimize anti-coagulant use in patients with non-valvular atrial fibrillation, has recently been introduced. The safety and efficacy of new-generation WATCHMAN FLX, as compared to conventional WATCHMAN 2.5, remain unknown. Methods: Consecutive patients who received percutaneous left atrial appendage closure using the WATCHMAN system in our institute, between June 2020 and December 2021, were retrospectively analyzed. Safety and efficacy during the 45-day observational period were compared between the two devices. Results: A total of 93 patients (73.0 ± 7.3 years old, 63 men) who received WATCHMAN FLX (*n* = 44) or WATCHMAN 2.5 (*n* = 49) were included. The device implant success rate was 100% in the FLX device group and 98% in the 2.5 device group. There were no procedure-related complications in the FLX group, and one non-relevant pericardial effusion in the 2.5 device group. During the 45-day observational period, there were no procedure-related adverse events. No patients in the FLX group had a peri-device leak >3 mm, whereas two patients in the 2.5 device group had a peri-device leak >3 mm. Anti-coagulants could be terminated in most of the patients (85% versus 88%; *p* = 0.68). Conclusions: Percutaneous left atrial appendage closure using new-generation WATCHMAN FLX seemed to be as safe and effective as conventional WATCHMAN 2.5 during the short-term observational period.

## 1. Background

The existence of non-valvular atrial fibrillation (NVAF) increases the risk of cardiogenic embolisms, most of which occur from the left atrial appendage [1,2]. Anti-coagulation therapy, using warfarin or direct oral anti-coagulant, is widely used to prevent embolisms [3]. The unsolved issues are poor adherence and an incremental risk of bleeding complications [4].

Several innovative devices that close the left atrial appendage to prevent thrombus formation have been created [5,6,7]. Of these, the WATCHMAN system (Scientific, Boston, MA, USA) has been demonstrated to be non-inferior to warfarin therapy in preventing cardiogenic embolism among NVAF patients with a high bleeding risk in the PROTECT AF trial and the PREVAIL trial [5,6]. The incidence of procedure-related severe complications, including cardiac tamponade, was relatively high during the early inclusion phase of the PROTECT AF trial. The incidence of such severe complications decreased during the study’s late inclusion phase. There are several potential pitfalls to be aware of to ensure successful left atrial appendage closure using the WATCHMAN system [8,9]. Recently, the EVOLUTION registry reported that the implantation success rate had improved, and procedure-related complications were lower, compared to the previous PROTECT-AF trial and PREVAIL trial [10].

**Figure 1:**
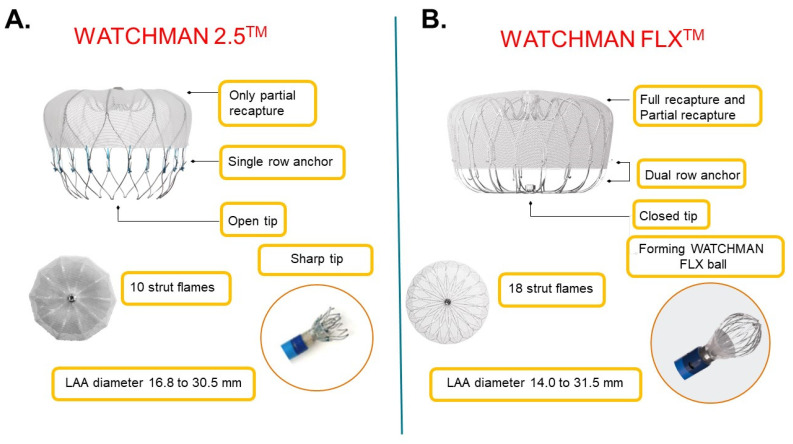
Profile comparison between WATCHMAN 2.5 (**A**) and WATCHMAN FLX (**B**).

The SALUTE trial reported on the safety of the procedure performed on 42 Japanese patients, and percutaneous left atrial appendage closure using the conventional WATCHMAN 2.5 system was reimbursed in Japan in 2019 (Figure 1A) [11]. The PINNACLE FLX trial demonstrated the safety of new-generation WATCHMAN FLX (Figure 1B), which was reimbursed in the USA in 2020 [12]. In this study, the incidence of serious device-related complications was only 0.5%, whereas the successful device implant rate was 98.8% [12]. According to the results of this trial, WATCHMAN FLX was also reimbursed in Japan in 2021. WATCHMAN FLX should be safer and more feasible than the conventional WATCHMAN 2.5, given that the FLX device was developed to overcome various drawbacks of the conventional device, as detailed in Figure 1A,B. In this preliminary study, we compared the short-term safety and efficacy between WATCHMAN FLX and WATCHMAN 2.5 in Japanese real-world daily practice. 

## 2. Methods

### 2.1. Patient Selection

All consecutive patients with NVAF who received percutaneous left atrial appendage closure using the WATCHMAN system in our institute between June 2020 and December 2021 were included in this retrospective study, using registry data. NVAF was defined as atrial fibrillation without moderate and severe mitral stenosis or any mechanical prosthetic heart valve. WATCHMAN 2.5 was used between June 2020 and June 2021. WATCHMAN FLX was used between July 2021 and December 2021, according to the institutional protocol. 

The indication of left atrial appendage closure accorded with the Japanese Circulation Society guidelines [13]. Candidates were those with NVAF who had high risks of experiencing systemic embolisms, and were highly recommended to receive anti-coagulation therapy, according to the CHADS_2_ score and CHA_2_DS_2_-VASc score [14]. Furthermore, the candidates had to satisfy either of the following: (1) those with HAS-BLED score ≥3 points, (2) those with multiple histories of trauma due to falling, (3) those with cerebral amyloid angiopathy, (4) those who had required multiple anti-platelets for over one year, and (5) those with histories of major bleeding assigned to BARC type 3–5. 

The study was approved by the institutional ethical board (R2020077) and informed consent was obtained from all patients before the listing. 

### 2.2. Procedures

Percutaneous left atrial appendage closure was performed under general anesthesia using angiography and transesophageal echocardiography supports, according to the standard procedure. All procedures were performed by the certified operator. Intra-procedure echocardiography was performed by the certified echocardiologist, who had completed a specific training program for the procedure. 

### 2.3. Post-Procedure Management

Post-procedural anti-thrombotic therapy was in accordance with the recommended regimen. Following the procedure, anti-coagulation therapy, using warfarin or direct anti-coagulant, and anti-platelet therapy, using one of 3 agents (aspirin, clopidogrel, and prasugrel), were performed for 45 days, after which transesophageal echocardiography was performed. Then, anti-coagulation therapy was terminated and dual anti-platelet therapy was initiated, unless major leak or device-related thrombus were found. Dual anti-platelet therapy was down-graded to single anti-platelet therapy six months later. 

The detailed medical therapy regimen was eventually at the discretion of the attending physicians, considering the risks of bleeding and thrombosis. 

### 2.4. Study Outcome

Procedure-related events were counted during the procedure and 1 week post procedure, or until the index discharge. Procedure-related events were defined as death, cerebrovascular events, systemic embolism, air embolism, bleeding, pericardial effusion, device embolization, and acute kidney injury. Of these, major procedure-related events were death, cerebrovascular events, systemic embolism, bleeding assigned to BARC 3–5, pericardial effusion, device embolization, and acute kidney injury. 

The events that occurred within 45 days were defined as death, cardiovascular death, cardiovascular event, systemic embolism, bleeding, myocardial infarction, ischemic stroke, hemorrhagic stroke, transient ischemic attack, and bleeding. 

### 2.5. Statistical Analysis

Continuous variables were expressed as mean and standard deviation, and were compared between the two groups using an unpaired *t*-test. Categorical variables were expressed as numbers and percentages, and were compared between the two groups using Fischer’s exact test. A value of *p* < 0.05 was considered statistically significant. Statistical analyses were performed using SPSS Statistics 24 (SPSS Inc., Armonk, IL, USA).

## 3. Results

### 3.1. Baseline Characteristics

A total of 93 patients (73.0 ± 7.3 years old, 63 men) were included (Table 1). All the patients had NVAF and satisfied the indication of percutaneous left atrial appendage closure using WATCHMAN 2.5 (*n* = 49) or WATCHMAN FLX (*n* = 44). The CHADS_2_ score was 3.3 ± 1.4, CHA_2_DS_2_-VASc score was 4.9 ± 1.5, and HAS-BLED score was 3.0 ± 0.9. Almost half of the patients had a history of bleeding. Almost all the patients received anti-coagulation and/or anti-platelet therapy at baseline. 

There were several statistical differences in the baseline characteristics between the two device groups (Table 1). The FLX group had a higher age and a lower HAS-BLED score, as well as a lower incidence of bleeding history (*p* < 0.01 for all). The FLX group preferred direct oral anti-coagulant to warfarin. 

### 3.2. Procedure Data

Almost all the procedures were successful, except for one patient who received WATCHMAN 2.5 (Table 2). In this patient, the left atrial appendage had a large ostium diameter, but not enough depth to implant the device. 

**Figure 2:**
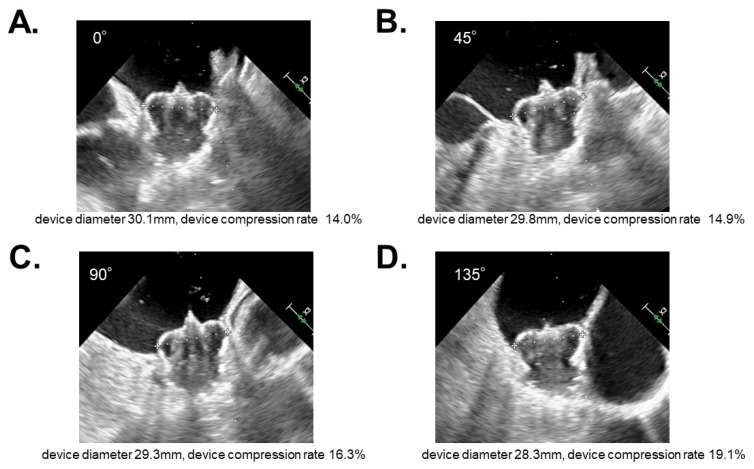
A representative case of WATCHMAN 2.5 (device size 33 mm) showing a variety of device compression rates observed by transesophageal echocardiography at 0 degrees (**A**), 45 degrees (**B**), 90 degrees (**C**), and 135 degrees (**D**).

The size of the left atrial appendage was not different between the two groups. A variety of device sizes were used—between 21 and 33 mm for the 2.5 device, and between 20 and 35 mm for the FLX device. The most frequently used device sizes in the 2.5 group and the FLX group were 33 mm and 31 mm, respectively. The standard deviation of the device compression rates at four angles (0, 45, 90, and 135 degrees) were lower in the FLX group than in the 2.5 group (*p* < 0.01; Figure 2A–D). In other words, the implanted FLX device was more orbicular than the 2.5 device. There were no peri-device leaks in either group. 

The dominant medication at the index discharge was single anti-platelet and anti-coagulant in both groups. Of these, the FLX group preferred direct oral anti-coagulant to warfarin. 

### 3.3. Echocardiographic Follow-Up Data

The follow-up data were obtained at 45 days following the procedures (Table 3). The follow-up was performed in all patients, except for the following two: one patient in whom WATCHMAN 2.5 implantation had failed, and another patient with WATCHMAN FLX who died due to procedure-unrelated myocardial infarction. 

**Figure 3:**
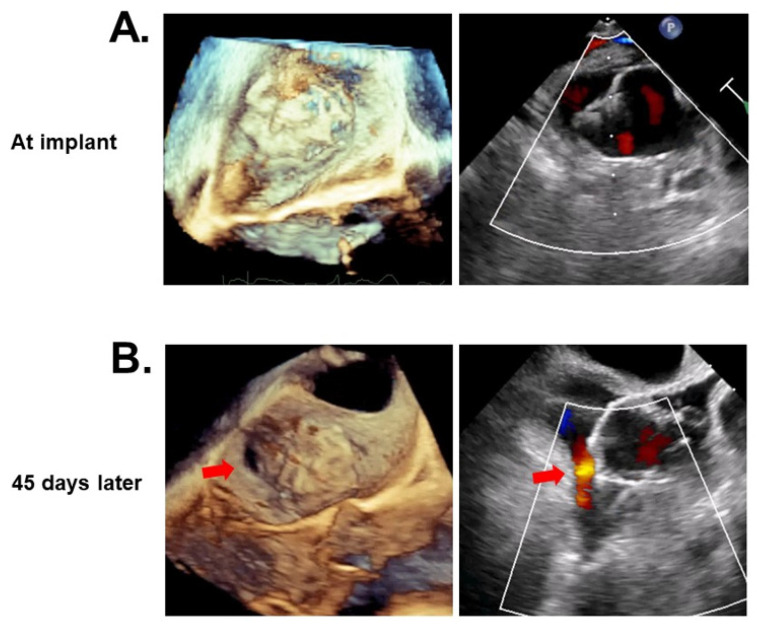
A representative case of a major peri-device leak at 45 days following WATCHMAN 2.5 implantation. At the time of device implantation (**A**) and at the 45-day follow-up with a major peri-device leak (red arrows; (**B**)).

Transesophageal echocardiography was performed in most of the patients. The standard deviation of the device compression rates at four angles was not different between the two device groups (*p* = 0.60). The incidences of peri-device leak were not different between the two device groups (*p* = 0.43). Most of them were within 3 mm. There was one patient with a 3–5 mm leak and another patient with a >5 mm leak in the 2.5 group (Figure 3A,B). Only one patient had device-related thrombosis following WATCHMAN 2.5 implantation. The prevalence of anti-coagulant termination was not different between the two device groups (85% versus 88%; *p* = 0.68). 

### 3.4. Clinical Events

**Figure 4:**
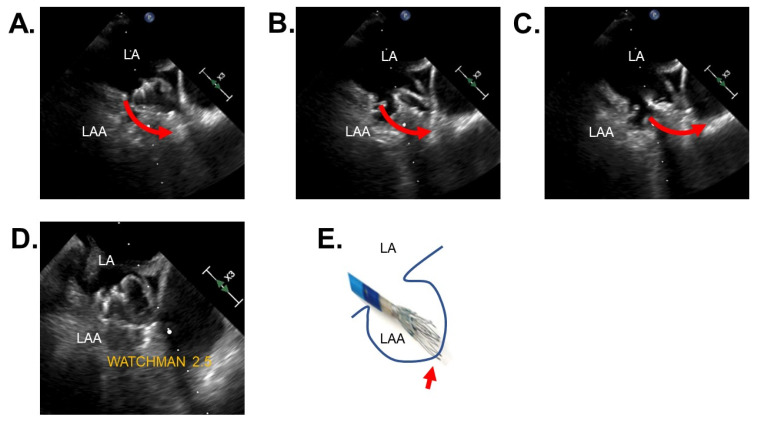
A representative case of left atrial appendage perforation and non-relevant pericardial effusion during WATCHMAN 2.5 implantation. (**A**) Immediately before perforation; during recapture; (**B**) a tip of WATCHMAN 2.5 device moved towards distal of left atrial appendage wall; (**C**) a tip of WATCHMAN device perforated the wall of left atrial appendage; (**D**) immediate closure of left atrial appendage by the device; (**E**) illustrations of the left atrium appendage and catheter. Red arrows indicate the direction of the catheter. A red arrow indicates the perforation site. LA, left atrium; LAA, left atrial appendage.

Table 4 summarizes the clinical outcomes, other than the echocardiographic findings, during the 45-day observational period. During the procedure, there was only one patient who had a procedure-related event, which was clinically non-relevant pericardial effusion during WATCHMAN 2.5 implantation (Figure 4). The patient was an 83-year-old woman with a history of cerebral embolism, hypertension, heart failure, post-trans-catheter aortic valve implantation, and on independent hemodialysis. She was taking single anti-platelet and direct oral anti-coagulant. Her HASBLED score was three points and her CHA_2_DS_2_VASc was six points. The wall of her left atrial appendage might have been injured by the distal tip during partial device recapture. The amount of effusion was small, with maintained hemodynamics, given the immediate closure of the left atrial appendage. 

There was only one patient who had a procedure-unrelated event, which was major bleeding that required blood transfusion, triggered by the concomitantly performed transcatheter mitral valve repairmen in the 2.5 device group.

Following the index discharge, there was only one patient who died due to myocardial infarction following WATCHMAN FLX implantation. The complication was eventually demonstrated not to be associated with the procedure. There were no cerebrovascular events in either group. There were five bleeding events, as follows: three in the 2.5 group and two in the FLX group (*p* = 0.72). 

## 4. Discussion

In this retrospective study using registry data, we compared safety and efficacy during a 45-day observational period, following percutaneous left atrial appendage closure using WATCHMAN 2.5 or WATCHMAN FLX. 

The incidence of procedure-related complications was low in the two device groups. Of note, there was no pericardial effusion, one of the fatal complications, in the FLX group. One patient with WATCHMAN 2.5 had non-relevant pericardial effusion. The 45-day incidence of bleeding and thrombosis was also low in the two device groups. The degree and incidence of peri-device leak at the 45-day follow-up were acceptable in all the patients (a patient with WATCHMAN 2.5 had >5 mm of leak). Anti-coagulants could be terminated in most patients at day 45. 

### 4.1. Innovation of New-Generation WATCHMAN FLX

The safety and efficacy of the left atrial appendage closure device, using the conventional WATCHMAN 2.5, were reported in two major randomized control trials [5,8]. The incidence of procedure-related complications, including pericardial effusion, device embolization, peri-device leak, and device-related thrombosis, was not satisfactory, particularly during the study’s early term. 

WATCHMAN FLX was innovated as a new-generation device to minimize these complications. The PINNACLE FLX trial demonstrated, for the first time, its safety and efficacy [1,4]. Several small-sized studies also reported the feasibility of WATCHMAN FLX implantation procedures, with an acceptably low complication rate [15,16,17]. Left atrial appendage closure with the WATCHMAN system has just been introduced in Japan, and there has been no study reporting the clinical outcomes following WATCHMAN FLX implantation.

### 4.2. Procedure-Related Complication

In this study, although preliminary studies and data were limited, we observed a low and clinically acceptable incidence of device-related complications during WATCHMAN FLX implantation in real-world practice. Of note, there was no pericardial effusion or device-related thrombosis during the implantation procedure. On the contrary, one patient had procedure-related pericardial effusion during WATCHMAN 2.5 implantation, although we cannot completely deny the procedure-unrelated spontaneous pericardial effusion in the high-risk cohorts (Figure 4). Such a complication might have been prevented by using WATCHMAN FLX, in which distal tines are folded back, forming a “flex ball”.

**Figure 5:**
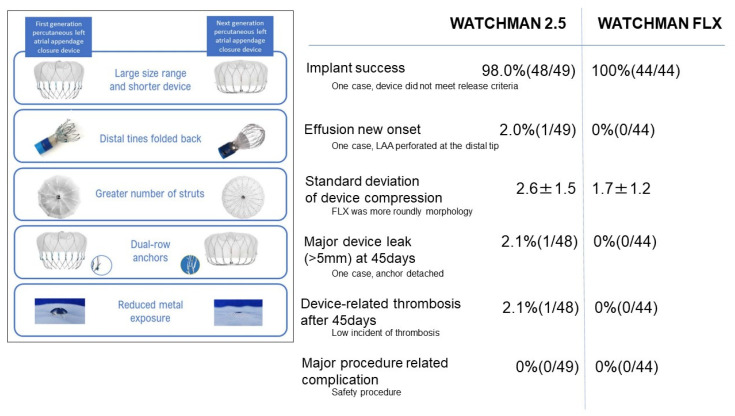
Summary of comparison between WATCHMAN FLX and WATCHMAN 2.5 in profiles and major outcomes.

We summarize the differences in device profile and major clinical outcomes between the two devices in Figure 5. As compared to the conventional WATCHMAN 2.5, the larger size range and shorter device size promise a high implant success rate. Distal tines are folded back, which may prevent cardiac wall injury. A greater number of struts and dual-row anchors may further minimize peri-device leak and device embolization. Reduced metal exposure may further prevent thrombus formation. 

Given the low incidence of these complications in the conventional 2.5 device group, there were no statistically significant advantages in the FLX group. Nevertheless, as shown in Figure 5, the incidences of these device-related major complications were all zero in the FLX group. A longer-term study, including a larger-scale cohort, is warranted to statistically demonstrate the advantage of WATCHMAN FLX over the conventional device. 

### 4.3. Short-Term Outcomes

At the 45-day follow-up, the incidences of peri-device leak were statistically not different between the two devices, but one patient in the 2.5 device group had a >5 mm major leak. In this patient, one of the anchors was detached and device malposition seemed to have occurred (Figure 3). We continued anti-coagulants after the 45-day follow-up in this patient, to prevent thrombus formation. The impact of the dual-row anchors system, as well as the more rounded morphology, in the FLX device in preventing device malposition and peri-device leak would require further long-term observation. 

A few patients had bleeding events during the 45-day follow-up period. Medication generally followed the guideline-recommended regimen. Further studies are warranted to establish a more sophisticated medication strategy to minimize the mid- to long-term incidence of bleeding/thrombosis. 

### 4.4. Limitations

The sample size was moderate. There were several statistically significant differences in baseline characteristics between the two devices, which might have affected the clinical outcomes. All the devices implanted in Japan from July 2021 were FLX. In other words, all the FLX devices in this study were implanted after the 2.5 device implantations. We cannot deny the bias of the learning curve. These differences might have affected the clinical outcomes following the implantation of devices, and we did not adjust for these potential confounders. The observational period was just 45 days following the device implantation, given that the device was reimbursed very recently in Japan. Given the small event number, we cannot conclude any “similarity” between the two devices, despite statistical non-significance. Nevertheless, it might be hypothesized that the newly introduced WATCHMAN FLX might be a promising next-generation device that is as safe and effective as a conventional WATCHMAN 2.5 device. A further large-scale long-term comparative study is warranted to assess the clinical feasibility of WATCHMAN FLX. 

## 5. Conclusions

Percutaneous left atrial appendage closure using new-generation WATCHMAN FLX seemed to be as safe and effective as conventional WATCHMAN 2.5 during the short-term observational period. Further longer-term observational studies, including larger-scale cohorts, are warranted to validate our findings and establish a more sophisticated percutaneous left atrial appendage closure strategy.

## Figures and Tables

**Table 1:** Baseline characteristics.

	Total (*n* = 93)	WATCHMAN 2.5 (*n* = 49)	WATCHMAN FLX (*n* = 44)	*p* Value
Demographics				
Sex, male	63 (68)	36 (74)	27 (61)	0.21
Age (years)	73.0 ± 7.3	76.4 ± 7.4	80.8 ± 6.6	<0.01 *
Body mass index (kg/m^2^)	23.3 ± 3.5	24.0 ± 3.2	22.5 ± 3.8	0.049 *
Body surface area (m^2^)	1.58 ± 0.17	1.62 ± 0.17	1.54 ± 0.16	0.035 *
Comorbidity				
Heart failure	56 (60)	28 (57)	28 (64)	0.53
New York Heart Association class II-IV	49 (53)	22 (45)	27 (61)	0.12
Hypertension	75 (81)	40 (82)	35 (80)	0.82
Diabetes mellitus	28 (30)	17 (35)	11 (25)	0.31
Prior stroke or transient ischemia attach	49 (53)	29 (59)	20 (45)	0.19
Prior ischemic stroke	35 (38)	20 (41)	15 (34)	0.51
Prior hemorrhagic stroke	13 (14)	9 (18)	4 (9)	0.20
Prior transient ischemic attack	5 (5)	4 (8)	1 (2)	0.20
Prior thromboembolic events	20 (22)	13 (27)	7 (16)	0.22
Hyperlipidemia	45 (48)	23 (46)	22 (50)	0.77
Coronary artery disease	30 (32)	17 (35)	13 (30)	0.60
Chronic obstructive pulmonary disease	2 (2)	0 (0)	2 (5)	0.16
Peripheral arterial disease	9 (10)	8 (16)	1 (2)	0.018 *
Chronic dialysis	22 (24)	17 (34)	5 (11)	<0.01 *
Paroxysmal atrial fibrillation	34 (37)	20 (41)	14 (32)	0.37
Prior intervention				
Prior myocardial infarction	8 (9)	6 (12)	2 (5)	0.19
Prior percutaneous coronary intervention	29 (31)	17 (35)	12 (27)	0.45
Prior coronary artery bypass grafting	6 (7)	5 (10)	1 (2)	0.12
Scores				
CHADS_2_ score	3.3 ± 1.4	3.2 ± 1.4	3.4 ± 1.4	0.54
CHA_2_DS_2_-VASc score	4.9 ± 1.5	4.9 ± 1.6	4.9 ± 1.4	0.76
HAS-BLED score	3.0 ± 0.9	3.2 ± 1.0	2.8 ± 0.8	<0.01 *
History of relevant bleeding				
Total	44 (47)	29 (59)	15 (34)	<0.01 *
Intracranial	21 (22)	11 (22)	10 (22)	0.98
Gastrointestinal	18 (19)	13 (27)	5 (11)	0.06
Hematuria	3 (3)	1 (2)	2 (4)	0.35
Respiratory	2 (2)	2 (4)	0 (0)	0.16
Epistaxis	2 (2)	2 (4)	0 (0)	0.16
Transthoracic echocardiography				
Left atrial diameter (mm)	43.7 ± 8.5	47.4 ± 9.2	46.5 ± 7.7	0.58
Left atrial volume index (ml/m^2^)	59.0 ± 23.4	63.3 ± 26.5	54.3 ± 18.8	0.07
Left ventricular end-diastolic diameter (mm)	47.9 ± 7.6	48.6 ± 8.1	47.2 ± 6.8	0.40
Left ventricular ejection fraction (%)	58.9 ± 13.5	56.6 ± 12.2	61.4 ± 14.6	0.09
Anti-platelet/anti-coagulant therapy at baseline				
None	1 (1)	0 (0)	1 (2)	0.32
Any SAPT	3 (3)	0 (0)	3 (7)	0.08
Any DAPT	3 (3)	1 (2)	2 (5)	0.50
Any single anti-coagulant therapy	55 (59)	28 (57)	27 (61)	0.68
Warfarin	11 (12)	9 (18)	2 (5)	0.035 *
DOAC	44 (47)	19 (39)	25 (57)	0.08
Any SAPT and anti-coagulant therapy	31 (33)	20 (41)	11 (25)	0.11
Any SAPT and Warfarin	13 (14)	12 (24)	1 (2)	<0.01 *
Any SAPT and DOAC	18 (19)	8 (16)	10 (23)	0.44
Any DAPT and anti-coagulant (triple therapy)	0 (0)	0 (0)	0 (0)	-

SAPT, single anti-platelet therapy; DAPT, dual anti-platelet therapy; DOAC, direct oral anti-coagulants. * *p* < 0.05. Continuous variables were compared by unpaired *t*-test. Categorical variables were compared by Fischer’s exact test.

**Table 2:** Procedural characteristics and post-procedural medications.

	WATCHMAN 2.5 (*n* = 49)	WATCHMAN FLX (*n* = 44)	*p* Value
General procedure data			
Procedure success	48 (98)	44 (100)	0.25
General anesthesia	49 (100)	44 (100)	1.0
Transesophageal echocardiography	49 (100)	44 (100)	1.0
Concomitant procedure	10 (20)	6 (14)	0.66
Procedure-related data			
Anesthesia time (min)	127 ± 31	127 ± 39	0.91
Fluoroscopy duration (min)	18 ± 10	17 ± 12	0.82
Procedure time (min)	63 ± 18	55 ± 20	0.047 *
Contrast volume (ml)	69 ± 23	76 ± 38	0.32
Left atrial appendage ostium diameter			
0 degree (mm)	20.9 ± 3.8	21.3 ± 4.0	0.60
45 degrees (mm)	19.4 ± 3.5	20.6 ± 3.9	0.13
90 degrees (mm)	20.1 ± 3.7	21.5 ± 4.0	0.09
135 degrees (mm)	23.0 ± 4.0	23.0 ± 3.9	0.98
Device size			
WATCHMAN 2.5 21/24/27/30/33 mm	2/1/11/11/23	-	-
WATCHMAN FLX 20/24/27/31/35 mm	-	2/4/12/14/11	-
Device compression rate			
0 degree (%)	16.8 ± 7.0	15.2 ± 5.2	0.22
45 degrees (%)	18.6 ± 5.8	15.4 ± 5.5	<0.01 *
90 degrees (%)	17.3 ± 6.7	14.3 ± 4.7	0.018 *
135 degrees (%)	14.4 ± 5.8	14.8 ± 5.7	0.72
Standard deviation of device compression at 4 angles	2.6 ± 1.5	1.7 ± 1.2	<0.01 *
Peri-device leak			
<3 mm	0 (0)	0 (0)	-
3–5 mm	0 (0)	0 (0)	-
>5 mm	0 (0)	0 (0)	-
Medications at index discharge			
None	0 (0)	0 (0)	-
Any SAPT	0 (0)	0 (0)	-
Any DAPT	0 (0)	0 (0)	-
Any single anti-coagulant therapy	8 (16)	9 (20)	0.6
Warfarin	1 (2)	0 (0)	0.35
DOAC	7 (15)	9 (20)	0.44
Any SAPT and anti-coagulant therapy	41 (85)	35 (80)	0.41
Any SAPT and Warfarin	22 (46)	3 (6)	<0.01 *
Any SAPT and DOAC	19 (40)	32 (72)	<0.01 *
Any DAPT and anti-coagulant (triple therapy)	0 (0)	0 (0)	-

Abbreviations as of Table 1. * *p* < 0.05. Continuous variables were compared by unpaired t-test. Categorical variables were compared by Fischer’s exact test.

**Table 3:** Transesophageal echocardiography findings and medications at 45-day follow-up.

	WATCHMAN 2.5 (*n* = 48)	WATCHMAN FLX (*n* = 43)	*p* Value
Transesophageal echocardiography			
Procedure completion	45 (93)	39 (91)	0.61
Device compression rate			
0 degree (%)	13.2 ± 6.6	12.4 ± 5.4	0.56
45 degrees (%)	15.1 ± 6.5	13.7 ± 6.7	0.36
90 degrees (%)	14.9 ± 6.2	12.1 ± 5.5	0.05
135 degrees (%)	12.7 ± 5.9	13.0 ± 5.9	0.80
Standard deviation of device compression at 4 angles	2.8 ± 1.1	2.9 ± 1.3	0.60
Peri-device leak			
<3 mm	8 (18)	6 (15)	0.77
3–5 mm	1 (2)	0 (0)	0.36
>5 mm	1 (2)	0 (0)	0.36
Device-related thrombosis	1 (2)	0 (0)	0.36
Medications			
None	0 (0)	0 (0)	-
Any SAPT	5 (10)	6 (14)	0.61
Any DAPT	36 (75)	32 (74)	0.95
Any single anti-coagulant therapy	6 (13)	3 (7)	0.38
Warfarin	0 (0)	0 (0)	-
DOAC	6 (13)	3 (7)	0.38
Any SAPT and anti-coagulant therapy	1 (2)	2 (5)	0.50
Any SAPT and Warfarin	1 (2)	0 (0)	0.35
Any SAPT and DOAC	0 (0)	2 (5)	0.16
Any DAPT and anti-coagulant (triple therapy)	0 (0)	0 (0)	-

Abbreviations as of Table 1. Continuous variables were compared by unpaired *t*-test. Categorical variables were compared by Fischer’s exact test. One patient in the WATCHMAN 2.5 group was excluded due to implant failure, and another patient in the WATCHMAN FLX group was excluded due to expiration following the index discharge.

**Table 4:** Clinical events at 45-day follow-up.

	WATCHMAN 2.5 (*n* = 49)	WATCHMAN FLX (*n* = 44)	*p* Value
Procedure related events	1 (2)	0 (0)	0.35
Major procedure related complication	0 (0)	0 (0)	-
Death	0 (0)	0 (0)	-
Cerebrovascular events	0 (0)	0 (0)	-
Systemic embolism	0 (0)	0 (0)	-
Air embolism	0 (0)	0 (0)	-
Any bleeding	0 (0)	0 (0)	-
Minor bleeding BARC 1–2	0 (0)	0 (0)	-
Major bleeding BARC 3–5	0 (0)	0 (0)	-
Pericardial effusion new onset	1 (2)	0 (0)	0.35
Clinically non-relevant	1 (2)	0 (0)	0.35
Clinically relevant	0 (0)	0 (0)	-
Vascular access site complication	0 (0)	0 (0)	-
Acute kidney injury	0 (0)	0 (0)	-
Non procedure related events			
Death	0 (0)	0 (0)	-
Cardiovascular death	0 (0)	0 (0)	-
Cardiovascular event	0 (0)	0 (0)	-
Systemic embolism	0 (0)	0 (0)	-
Any bleeding	1 (2)	0 (0)	0.35
Minor bleeding BARC 1–2	0 (0)	0 (0)	-
Major bleeding BARC 3–5	1 (2)	0 (0)	0.35
All clinical events at 45-day follow-up			
Death	0 (0)	1 (2)	0.32
Cardiovascular death	0 (0)	1 (2)	0.32
Cerebrovascular event	0 (0)	0 (0)	-
Stroke	0 (0)	0 (0)	-
Ischemic stroke	0 (0)	0 (0)	-
Hemorrhagic stroke	0 (0)	0 (0)	-
Transient ischemic attack	0 (0)	0 (0)	-
Systemic embolism	0 (0)	0 (0)	
Myocardial infarction	0 (0)	1 (2)	0.32
Any bleeding	3 (6)	2 (5)	0.74
Minor bleeding BARC 1–2	0 (0)	1 (2)	0.32
Major bleeding BARC 3–5	3 (6)	1 (2)	0.37
Pericardial effusion new onset	0 (0)	0 (0)	-

Variables were compared by Fischer’s exact test.

## Data Availability

Data are available from the corresponding authors upon reasonable requests.

## References

[1] Wolf P.A., Abbott R.D., Kannel W.B. (1991). Atrial fibrillation as an independent risk factor for stroke: The Framingham Study. Stroke.

[2] Blackshear J.L., Odell J.A. (1996). Appendage obliteration to reduce stroke in cardiac surgical patients with atrial fibrillation. Ann. Thorac. Surg..

[3] January C.T., Wann L.S., Calkins H., Chen L.Y., Cigarroa J.E., Cleveland J.C., Ellinor P.T., Ezekowitz M.D., Field M.E., Furie K.L. (2019). 2019 AHA/ACC/HRS Focused Update of the 2014 AHA/ACC/HRS Guideline for the Management of Patients with Atrial Fibrillation: A Report of the American College of Cardiology/American Heart Association Task Force on Clinical Practice Guidelines and the Heart Rhythm Society in Collaboration with the Society of Thoracic Surgeons. Circulation.

[4] Hart R.G., Pearce L., Aguilar M.I. (2007). Meta-analysis: Antithrombotic Therapy to Prevent Stroke in Patients Who Have Nonvalvular Atrial Fibrillation. Ann. Intern. Med..

[5] Holmes D.R., Kar S., Price M.J., Whisenant B., Sievert H., Doshi S.K., Huber K., Reddy V.Y. (2014). Prospective randomized evaluation of the Watchman Left Atrial Appendage Closure device in patients with atrial fibrillation versus long-term warfarin therapy: The PREVAIL trial. J. Am. Coll. Cardiol..

[6] Holmes D.R., Reddy V., Turi Z.G., Doshi S.K., Sievert H., Buchbinder M., Mullin C.M., Sick P. (2009). Percutaneous closure of the left atrial appendage versus warfarin therapy for prevention of stroke in patients with atrial fibrillation: A randomised non-inferiority trial. Lancet.

[7] Osmancik P., Herman D., Neuzil P., Hala P., Taborsky M., Kala P., Poloczek M., Stasek J., Haman L., Branny M. (2020). Left Atrial Appendage Closure Versus Direct Oral Anticoagulants in High-Risk Patients with Atrial Fibrillation. J. Am. Coll. Cardiol..

[8] Reddy V.Y., Holmes D., Doshi S.K., Neuzil P., Kar S. (2011). Safety of percutaneous left atrial appendage closure: Results from the watchman left atrial appendage system for embolic protection in patients with AF (PROTECT AF) clinical trial and the continued access registry. Circulation.

[9] Holmes D.R., Doshi S.K., Kar S., Price M.J., Sanchez J.M., Sievert H., Valderrabano M., Reddy V.Y. (2015). Left Atrial Appendage Closure as an Alternative to Warfarin for Stroke Prevention in Atrial Fibrillation: A Patient-Level Meta-Analysis. J. Am. Coll. Cardiol..

[10] Boersma L.V., Ince H., Kische S., Pokushalov E., Schmitz T., Schmidt B., Gori T., Meincke F., Protopopov A., Betts T. (2017). Efficacy and safety of left atrial appendage closure with WATCHMAN in patients with or without contraindication to oral anticoagulation: 1-Year follow-up outcome data of the EWOLUTION trial. Heart Rhythm.

[11] Aonuma K., Yamasaki H., Nakamura M., Matsumoto T., Takayama M., Ando K., Hirao K., Goya M., Morino Y., Hayashida K. (2020). Efficacy and Safety of Left Atrial Appendage Closure with WATCHMAN in Japanese Nonvalvular Atrial Fibrillation Patients―Final 2-Year Follow-Up Outcome Data from the SALUTE Trial. Circ. J..

[12] Kar S., Doshi S.K., Sadhu A., Horton R., Osorio J., Ellis C., Stone J., Shah M., Dukkipati S.R., Adler S. (2021). Primary outcome evaluation of a next generation left atrial appendage closure device: Results from the PINNACLE FLX trial. Circulation.

[13] Nogami A., Kurita T., Kusano K., Goya M., Shoda M., Tada H., Naito S., Yamane T., Kimura M., Shiga T. (2022). JCS/JHRS 2021 Guideline Focused Update on Non-Pharmacotherapy of Cardiac Arrhythmias. Circ. J..

[14] Ono K., Iwasaki Y., Shimizu W., Akao M., Ikeda T., Ishii K., Inden Y., Kusano K., Kobayashi Y., Koretsune Y. (2022). JCS/JHRS 2020 Guideline on Pharmacotherapy of Cardiac Arrhythmias. Circ. J..

[15] Cruz-González I., Korsholm K., Trejo-Velasco B., Thambo J.B., Mazzone P., Rioufol G., Grygier M., Möbius-Winkler S., Betts T., Meincke F. (2020). Procedural and Short-Term Results with the New Watchman FLX left atrial appendage occlusion device. J. Am. Coll. Cardiol. Interv..

[16] Korsholm K., Samaras A., Andersen A., Møller Jensen J., Nielsen-Kudsk J.E. (2020). The Watchman FLX Device: First European Experience and Feasibility of Intracardiac Echocardiography to Guide Implantation. J. Am. Coll. Cardiol..

[17] Cruz-González I., Torres Saura F., Trejo-Velasco B., Antonio Fernández Díaz J., Fajardo Molina R., Del Valle-Fernández R., Moreno Terribas G., Martí Sánchez D., López-Mínguez J.R., Gomez-Blazquez I. (2022). Impact of operators experience on peri-procedural outcomes with Watchman FLX: Insights from the FLX-SPA registry. Int. J. Cardiol. Heart Vasc..

